# The Lumen of Human Intestinal Organoids Poses Greater Stress to Bacteria Compared to the Germ-Free Mouse Intestine: Escherichia coli Deficient in RpoS as a Colonization Probe

**DOI:** 10.1128/mSphere.00777-20

**Published:** 2020-11-11

**Authors:** Madeline R. Barron, Roberto J. Cieza, David R. Hill, Sha Huang, Veda K. Yadagiri, Jason R. Spence, Vincent B. Young

**Affiliations:** a Department of Microbiology and Immunology, University of Michigan Medical School, Ann Arbor, Michigan, USA; b Department of Internal Medicine, Division of Infectious Diseases, University of Michigan Medical School, Ann Arbor, Michigan, USA; c Department of Internal Medicine, Division of Gastroenterology, University of Michigan Medical School, Ann Arbor, Michigan, USA; d Department of Cell and Developmental Biology, University of Michigan Medical School, Ann Arbor, Michigan, USA; e Department of Biomedical Engineering, University of Michigan Medical School, Ann Arbor, Michigan, USA; University of Maryland School of Medicine

**Keywords:** RpoS, colonization, intestine, organoid, stress

## Abstract

Pluripotent stem-cell-derived human intestinal organoids (HIOs) are three-dimensional, multicellular structures that model a naive intestinal epithelium in an *in vitro* system. Several published reports have investigated the use of HIOs to study host-microbe interactions. We recently demonstrated that microinjection of the nonpathogenic Escherichia coli strain ECOR2 into HIOs induced morphological and functional maturation of the HIO epithelium, including increased secretion of mucins and cationic antimicrobial peptides. In the current work, we use ECOR2 as a biological probe to further characterize the environment present in the HIO lumen. We generated an isogenic mutant in the general stress response sigma factor RpoS and employed this mutant to compare challenges faced by a bacterium during colonization of the HIO lumen relative to the germ-free mouse intestine. We demonstrate that the loss of RpoS significantly decreases the ability of ECOR2 to colonize HIOs, although it does not prevent colonization of germ-free mice. These results indicate that the HIO lumen is a more restrictive environment to E. coli than the germ-free mouse intestine, thus increasing our understanding of the HIO model system as it pertains to studying the establishment of intestinal host-microbe symbioses.

**IMPORTANCE** Technological advancements have driven and will continue to drive the adoption of organotypic systems for investigating host-microbe interactions within the human intestinal ecosystem. Using E. coli deficient in the RpoS-mediated general stress response, we demonstrate that the type or severity of microbial stressors within the HIO lumen is more restrictive than those of the *in vivo* environment of the germ-free mouse gut. This study provides important insight into the nature of the HIO microenvironment from a microbiological standpoint.

## Abstract (precis)

Technological advancements have driven and will continue to drive the adoption of organotypic systems for investigating host-microbe interactions within the human intestinal ecosystem. Using E. coli deficient in the RpoS-mediated general stress response, we demonstrate that the type or severity of microbial stressors within the HIO lumen is more restrictive than those of the *in vivo* environment of the germ-free mouse gut. This study provides important insight into the nature of the HIO microenvironment from a microbiological standpoint.

## INTRODUCTION

The mammalian gastrointestinal tract is inhabited by a diverse community of microbes that play critical roles in host development and health, including facilitating the development and maturation of the intestinal epithelial barrier (1–3). The intestinal epithelium represents an important physical and biochemical interface through which host-microbe symbioses within the gut are established and maintained (4). Historically, the model systems available to study intestinal epithelial-microbe interactions consisted primarily of immortalized cell lines and animal models. For instance, germ-free mice have become the gold standard for investigating host and microbial responses during the establishment of defined bacterial populations within the microbe-naive gut (5–10). However, technological advancements have expanded the repertoire of systems available for studying host-microbe interactions at the intestinal interface. In this regard, stem-cell-derived human intestinal organoids (HIOs) have emerged as powerful tools to investigate epithelial structure and function following initial interactions with a range of bacterial species (11–13).

HIOs are three-dimensional, organotypic structures comprised of multiple types of differentiated epithelial cells surrounded by a supporting mesenchyme (13–16). Derived from embryonic or induced pluripotent stem cells, HIOs possess many aspects of the microbe-naive intestinal epithelium in an experimentally tractable, *in vitro* system (13, 15, 16). We recently reported that microinjection of ECOR2, a nonpathogenic strain of Escherichia coli originally isolated from a healthy individual (17), into the lumen of HIOs induced transcriptional, morphological, and functional changes in HIOs resulting in the maturation of the epithelium (14). These changes included increased expression of genes regulating epithelial tight junction proteins, increased production of mucins, and secretion of antimicrobial peptides (14). Of note, E. coli established a stable population within the HIO lumen for several days after microinjection, with preservation of HIO epithelial barrier integrity (14). Overall, this study highlighted the utility of HIOs for studying epithelial adaptation to the establishment of bacterial populations at the intestinal epithelial interface. However, despite this demonstrated utility of HIOs, most studies have focused on epithelial responses, whereas less has been done to understand the HIO microenvironment from a microbiological standpoint. In the current work, we used E. coli as a biological probe to better characterize the HIO luminal environment from the perspective of a bacterial colonizer.

E. coli adapts to its environment via the well-characterized “general stress response,” which is regulated by the stress response sigma factor RpoS (18–20). Upon exposure to environmental stressors, including nutrient limitation, oxidative stress, and low pH (19), RpoS alters global bacterial gene expression to switch the cell from a state of active growth to one of survival (19). While RpoS has been extensively studied in the context of laboratory-imposed environmental stress, it has also been shown to facilitate bacterial colonization of the gut. For example, RpoS is important for efficient intestinal colonization by several pathogenic bacterial species, including Vibrio cholerae, Salmonella enterica serovar Typhimurium, and the virulent E. coli strain O157:H7 (21, 22). In addition, it was previously reported that RpoS may play a role in the initial establishment of nonpathogenic E. coli within the streptomycin-treated mouse gut (23). Given the role of RpoS in colonization of the intestine, we leveraged this information to compare the challenges faced by a bacterium when colonizing the HIO lumen and the murine gut. Using an isogenic Δ*rpoS* mutant of E. coli strain ECOR2, we demonstrate that the loss of RpoS attenuates the ability of ECOR2 to colonize HIOs, although it does not prevent colonization of germ-free mice. Rather, the Δ*rpoS* mutant exhibits a fitness defect in the mouse gut only in the context of microbial competition. Our results suggest that relative to the *in vivo* environment of the germ-free mouse intestine, the HIO lumen provides a greater challenge to E. coli during colonization. These results increase our understanding of the HIO model system as it pertains to studying intestinal epithelial-microbe interactions.

## RESULTS

### Generation and *in vitro* characterization of an isogenic Δ*rpoS* mutant of E. coli strain ECOR2.

**FIG 1:**
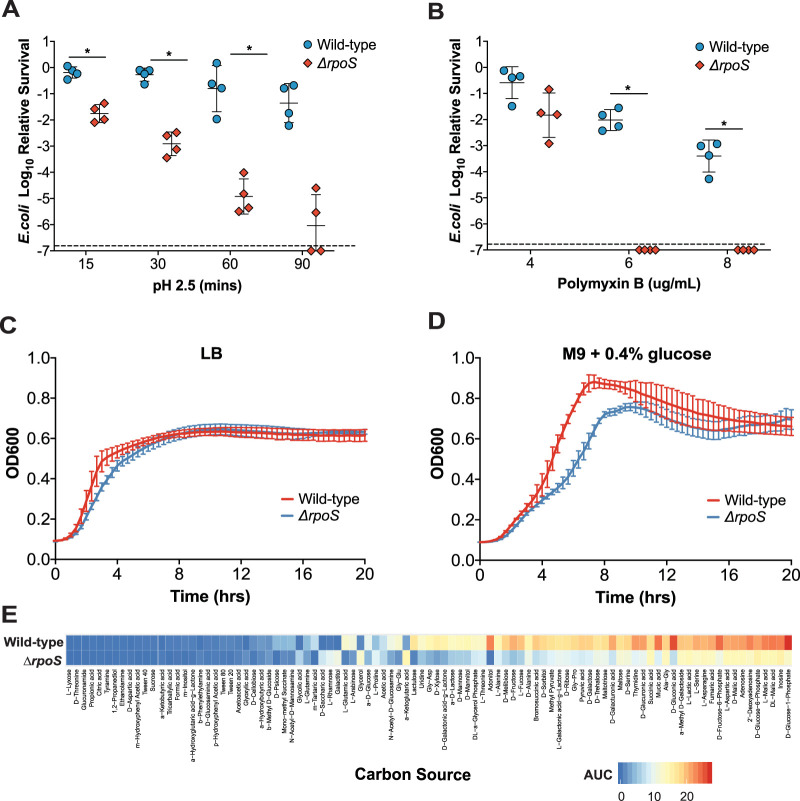
An isogenic Δ*rpoS* mutant of E. coli strain ECOR2 exhibits altered carbon metabolism and increased sensitivity to low pH, membrane stress, and nutrient limitation *in vitro*. (A and B) Wild-type ECOR2 and an isogenic Δ*rpoS* mutant were tested for survival after exposure to LB medium adjusted to pH 2.5 for 15, 30, 60, and 90 min and after 1 h of treatment with 4, 6, and 8 μg/ml polymyxin B. Relative survival was calculated by dividing the CFU from the treated samples by those of the untreated control for each E. coli strain. The dashed line indicates the limit of detection (*n* = 4 biological replicates per E. coli strain for each assay). Values were log transformed prior to analysis. Error bars represent standard deviations of the means. *, *P* < 0.05 by a Mann-Whitney U test or Welch’s unpaired *t* test depending on the data distribution. (C and D) Growth curves of wild-type ECOR2 and the Δ*rpoS* mutant in LB or M9 medium supplemented with 0.4% glucose were conducted at 37°C in a plate reader with shaking for 20 h. Error bars denote standard deviations of the means from at least 3 biological replicates per E. coli strain. (E) Growth of wild-type ECOR2 and the Δ*rpoS* mutant with 95 individual carbon sources using Biolog PM1 plates. The metabolic capacity of each strain, depicted as a heat map, is represented as the area under the curve (AUC) of OD_595_ values plotted over 24 h for each carbon source.

In this study, we used E. coli deficient in the RpoS-mediated stress response as a probe to better understand the HIO luminal environment from a microbiological standpoint. We generated an isogenic Δ*rpoS* mutant of E. coli strain ECOR2 (see Fig. S1A and B in the supplemental material) and first verified its phenotype by subjecting it to various *in vitro* stressors. RpoS is known to protect against exposure to low pH (19); thus, we tested the ability of the Δ*rpoS* mutant to survive incubation in LB broth adjusted to pH 2.5. As expected, the Δ*rpoS* mutant exhibited increased sensitivity to acid stress relative to the wild-type parent strain (Fig. 1A). Incubation for 1 h in LB broth containing 4, 6, or 8 μg/ml of polymyxin B, an antibiotic that binds lipopolysaccharide to destabilize bacterial membrane integrity (24), also revealed that the Δ*rpoS* mutant displayed a reduced ability to tolerate membrane stress compared to wild-type bacteria (Fig. 1B).

10.1128/mSphere.00777-20.1 FIG S1 Loss of *rpoS* expression in an isogenic Δ*rpoS* ECOR2 mutant. An isogenic Δ*rpoS* mutant of ECOR2 was generated by inserting the *neo* gene cassette (conferring kanamycin resistance) into the *rpoS* gene via lambda red recombination (see Materials and Methods). (A) Agarose gel of endpoint PCR products illustrating the insertion of the 1.4-kb *neo* cassette into the *rpoS* gene of Δ*rpoS* ECOR2 compared to the wild-type parent strain. (B) qRT-PCR analyses of *rpoS* gene expression in wild-type and Δ*rpoS* ECOR2 after 8 h of growth in LB medium. The relative expression level of *rpoS* was determined via the ΔΔ*C_T_* method using *rpoA* gene transcripts as controls. Error bars represent standard deviations from 3 technical replicates. Download FIG S1, EPS file, 2.0 MB. Copyright © 2020 Barron et al. 2020 Barron et al. This content is distributed under the terms of the Creative Commons Attribution 4.0 International license.

Previous studies have demonstrated that the loss of RpoS alters E. coli metabolism (25, 26). To this point, although the Δ*rpoS* mutant grew similarly to wild-type ECOR2 in LB broth (Fig. 1C), it exhibited a growth defect in minimal medium supplemented with glucose as a carbon source (Fig. 1D). Furthermore, a catabolic screen using Biolog PM1 (carbon nutrition) microplates revealed that the Δ*rpoS* mutant was defective in its ability to metabolize all 61 substrates that supported the growth of wild-type ECOR2 (Fig. 1E and Table S1). These data support that the loss of RpoS alters ECOR2 carbon metabolism and modulates growth depending on nutrient availability. Together, the results from these experiments confirmed the importance of RpoS for environmental adaptation by E. coli strain ECOR2. Thus, we used the Δ*rpoS* ECOR2 mutant to investigate the relative stress imposed by the HIO lumen on colonizing bacteria.

10.1128/mSphere.00777-20.2 TABLE S1 Area under the curve of OD_595_ values collected during wild-type and Δ*rpoS* ECOR2 mutant growth in 95 individual carbon sources using Biolog PM1 plates (see Fig. 1E in the main text). Download Table S1, DOCX file, 0.02 MB. Copyright © 2020 Barron et al. 2020 Barron et al. This content is distributed under the terms of the Creative Commons Attribution 4.0 International license.

### The HIO lumen is restrictive to colonization by Δ*rpoS* ECOR2 compared to the germ-free mouse gut.

**FIG 2:**
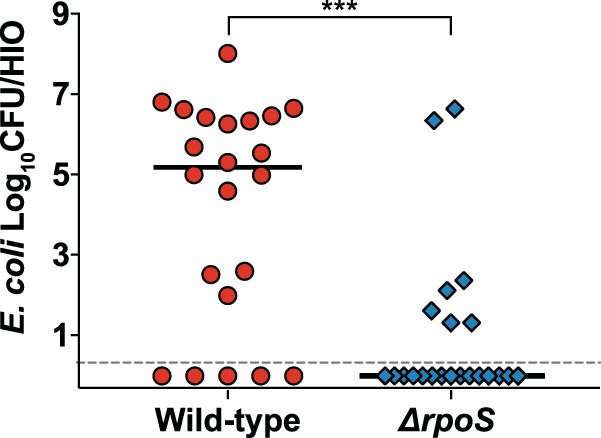
Loss of RpoS attenuates the ability of ECOR2 to colonize HIOs. Luminal CFU were obtained from individual HIOs 24 h after microinjection with ∼10^4^ CFU of either wild-type ECOR2 or its isogenic Δ*rpoS* mutant in PBS (*n* = 22 biological replicates per E. coli strain combined from five independent experiments). Error bars denote medians; the dashed line indicates the limit of detection. ***, *P* < 0.001 by a Mann-Whitney U test.

The luminal microenvironment of HIOs is altered following microbial colonization, including a reduction in luminal oxygen concentrations and the deployment of epithelial antimicrobial defense mechanisms (14). To determine whether these HIO responses pose a challenge to bacteria within the HIO lumen, wild-type ECOR2 or the isogenic Δ*rpoS* mutant was microinjected into individual HIOs, and bacteria were enumerated after 24 h. Comparing the luminal CFU obtained from HIOs microinjected with either strain, we found that the Δ*rpoS* mutant exhibited a significant defect in its ability to establish populations within HIOs compared to wild-type ECOR2 (Fig. 2).

**FIG 3:**
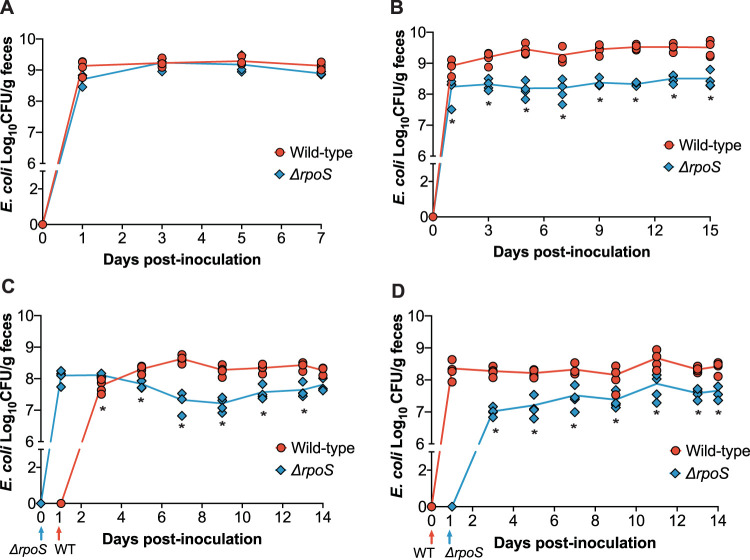
Δ*rpoS* ECOR2 exhibits a colonization defect in the germ-free mouse gut only in the context of microbial competition. Germ-free Swiss Webster mice were either monoassociated with 10^6^ to 10^7^ total CFU of wild-type or Δ*rpoS* mutant ECOR2 (A) or competitively inoculated with a 1:1 ratio of each strain together (B) or 24 h apart (C and D). At the indicated times, fecal samples were homogenized, diluted, and plated as described in Materials and Methods (*n* = 3 to 4 mice per time point). *, *P* < 0.05 by Mann-Whitney U tests. WT, wild type.

The stark colonization defect exhibited by the Δ*rpoS* mutant in HIOs prompted us to assess its colonization dynamics in a corresponding murine model. To do this, we colonized germ-free Swiss Webster mice with ∼10^7^ CFU of wild-type ECOR2 or the Δ*rpoS* mutant (Fig. 3A). Feces were collected and plated every other day for 7 days. Interestingly, in contrast to what we had observed in HIOs, the Δ*rpoS* mutant colonized just as well as wild-type ECOR2 during monoassociation of germ-free mice (Fig. 3A).

Within the intestine, bacteria must compete with one another for a limited number of available niches (27). Thus, we posited that the relative stress within the gut environment would increase in the context of microbial competition and thus could influence the ability of the Δ*rpoS* mutant to stably colonize the intestine. We specifically asked whether the colonization dynamics of the Δ*rpoS* mutant would change during competition with the wild-type strain. To address this question in a system where we could look at interstrain competition without competition from other microbes, we colonized germ-free mice with equal amounts of wild-type and Δ*rpoS* ECOR2 bacteria (∼10^6^ total CFU) and measured fecal levels of each strain over 15 days. While the Δ*rpoS* mutant was still able to colonize the gut, it exhibited a fitness defect during co-colonization with wild-type ECOR2 (Fig. 3B). We hypothesized that allowing the Δ*rpoS* mutant to establish within the germ-free mouse gut prior to the introduction of the wild-type strain would rescue this defect. To test this hypothesis, mice were colonized with the Δ*rpoS* mutant followed by the wild-type strain 24 h later, or vice versa (Fig. 3D and E). Feces were collected and plated over 14 days. We found that the Δ*rpoS* mutant displayed a competitive disadvantage regardless of whether it was introduced before (Fig. 3D) or after (Fig. 3E) the wild-type strain, thus indicating that the order of colonization does not matter.

## DISCUSSION

In this study, we used an isogenic Δ*rpoS* mutant of E. coli strain ECOR2 as a biological probe to investigate the HIO luminal environment compared to the germ-free mouse gut from the perspective of a bacterial colonizer. In E. coli and other Gram-negative bacteria, the ability to respond to diverse stressors in the environment, including within the gut, occurs via the RpoS-mediated stress response (18–23). Indeed, our data demonstrate that the loss of RpoS decreases the ability of E. coli strain ECOR2 to withstand membrane stress and exposure to low pH as well as alters its capacity to metabolize a broad range of carbon substrates. Importantly, we found that the loss of RpoS significantly decreased the ability of ECOR2 to colonize HIOs, though it did not prevent colonization of germ-free mice. These data indicate that the type or severity of microbial stressors within the HIO lumen is more restrictive than those of the *in vivo* intestinal environment.

There are likely a number of factors that shape the distinct environment of the HIO lumen. We have shown that the HIO epithelium produces antimicrobial peptides, which could be particularly stressful to microbes within the small, static luminal space (14). To this point, since there is no peristalsis and luminal flow within HIOs, this leads to a buildup of epithelial and microbe-derived waste within the HIO lumen (28). In this regard, technological advancements that establish steady-state liquid flow through the HIO lumen (28) may lessen or eliminate microbial stressors within the HIO microenvironment. Given our Biolog data demonstrating that the Δ*rpoS* mutant has a limited nutritional repertoire relative to wild-type ECOR2, another hypothesis is that the HIO lumen is more restricted in terms of nutrient availability than the murine intestine. Indeed, the mouse gastrointestinal tract contains a relatively rich nutrient pool that is replenished during regular feeding (29, 30). Furthermore, several studies have demonstrated that nutrient availability is the primary driver of E. coli colonization and adaptation within the murine gut (9, 31, 32). Thus, nutrient availability may explain the colonization dynamics observed *in vivo*, where the Δ*rpoS* mutant grew to wild-type levels during monoassociation of germ-free mice yet displayed a colonization defect in the context of microbial competition. The nutrient milieu of HIOs is relatively unknown, though it is presumably derived from the surrounding culture medium that has been further modified by the metabolic activities of the epithelium (33) and mesenchyme. Therefore, the diversity and concentration of substrates available for E. coli consumption within HIOs are potentially limited. Ultimately, we suspect that there are a number of conditions that together create the relatively hostile environment within HIOs. More extensive work is required to characterize the exact nature of the nutritional or epithelial-derived microbial stressors inherent to the HIO lumen.

Overall, we have demonstrated that, from a bacterial standpoint, HIOs possess a unique luminal environment relative to the germ-free mouse intestine. Our results indicate that the type or severity of microbe-perceived stress within the HIO lumen is more restrictive than that of the *in vivo* gut environment. As organotypic models continue to be employed for investigating intestinal host-microbe interactions, it is critical that we benchmark these systems so that experiments can be put into proper perspective. Moving forward, the results from this study will better inform when and how we use HIOs to investigate diverse aspects of host-microbe symbioses within the gut.

## MATERIALS AND METHODS

### Bacterial strains and plasmids.

Strains and plasmids are listed in Table 1. Escherichia coli strain ECOR2 (ATCC 35321) (17) was used in the present study. An isogenic Δ*rpoS* mutant was constructed by disruption of the ECOR2 *rpoS* gene via lambda red-mediated gene replacement with the aminoglycoside phosphotransferase gene (*neo*), which encodes resistance to kanamycin (34). The *neo* gene was amplified from the plasmid pKD4 (34) using primers MB001-F and MB001-R. The *neo* PCR product containing 50 bases upstream and downstream homologous to those of the *rpoS* gene was subsequently electroporated into ECOR2 containing plasmid pKD46, which contains the phage lambda Red recombinase gene cluster (34). Successful disruption of the *rpoS* gene was verified by endpoint PCR using primers MB002-F and MB002-R, Sanger sequencing was conducted with primers MB003-F and MB003-R, and real-time reverse transcription-quantitative PCR (qRT-PCR) was performed with primers rpoS-RT-F and rpoS-RT-R (see below and Fig. S1A and B in the supplemental material). Bacteria were grown aerobically in LB broth or on LB agar at 37°C. In all experiments, medium was supplemented with kanamycin sulfate (50 μg/ml) for growth of the Δ*rpoS* mutant.

**TABLE 1:** Bacterial strains, plasmids, and primers used in this study

E. coli strain, primer, or plasmid	Relevant characteristic(s) and/or sequence (5′–3′)	Reference
E. coli strains		
ECOR2	Nonpathogenic E. coli strain isolated from a healthy individual	17
ECOR2 Δ*rpoS*	ECOR2 *rpoS* disrupted with a *neo* cassette; Km^r^	This study

Primers		
MB001-F	ATGAGTCAGAATACGCTGAAAGTTCATGATTTAAATGAAGATGCGGAATT; for lambda red replacement	This study
MB001-R	TTACTCGCGGAACAGCGCTTCGATATTCAGCCCCTGCGTTTGCAGGATTT; for lambda red replacement	This study
MB002-F	ATGAGTCAGAATACGCTGAAAGTTC; forward primer to amplify *rpoS*; product length, 993 bp	This study
MB002-R	TTACTCGCGGAACAGCGCTTCGATA; reverse primer to amplify *rpoS*	This study
MB003-F	CCAGTTCAACACGCTTGCAT; forward primer for Sanger sequencing (123 bp upstream of *rpoS*); product length, 1.25 kb	This study
MB003-R	GTGCGTATGGGCGGTAATTT; reverse primer for Sanger sequencing (95 bp downstream of *rpoS*)	This study
rpoS-RT-F	CCTGGCCGATGAAAAAGAG; forward primer for qRT-PCR analysis of *rpoS* expression; product length, 81 bp	37
rpoS-RT-R	AACAGCCATTTGACGATGCTC; reverse primer for qRT-PCR analysis of *rpoS* expression	37
rpoA-F	ATGCAGGGTTCTGTGACAGA; forward primer to amplify *rpoA* for qRT-PCR; product length, 140 bp	38
rpoA-R	AGAATACGGCGCAGTGCGTT; reverse primer to amplify *rpoA* for qRT-PCR	38

Plasmids		
pKD46	Temp-sensitive Red+Gam-expressing plasmid; Ap^r^	34
pKD4	Template plasmid containing the *neo* gene template; Km^r^ Ap^r^	34

### qRT-PCR.

qRT-PCR was used to verify the loss of *rpoS* expression in the Δ*rpoS* ECOR2 mutant. Cultures of wild-type and Δ*rpoS* ECOR2 grown overnight were diluted 1:100 in sterile LB broth. After 8 h of incubation, 1 ml of each culture was added to 2 ml RNAprotect bacterial reagent (Qiagen) according to the manufacturer’s instructions. This time point was chosen because it correlates with entry into the stationary phase of growth (Fig. 1C and D), when RpoS is most abundant in the cell (19, 35). Samples were stored at −20°C until RNA extraction. RNA was extracted from each sample using the RNeasy minikit (Qiagen) and quantified with the Quant-IT RiboGreen RNA assay kit (Invitrogen). One microgram of RNA was reverse transcribed to cDNA with the QuantiTect reverse transcription kit (Qiagen) according to the manufacturer’s instructions. For qRT-PCR analyses, 20-μl reaction mixtures were prepared using the QuantiTect SYBR green PCR kit (Qiagen) and primers rpoS-RT-F and rpoS-RT-R. qRT-PCR was performed on a LightCycler96 qPCR machine (Roche) with 45 cycles of 94°C for 15 s, 53°C for 30 s, and 72°C for 30 s. The relative expression level of *rpoS* was determined via the ΔΔ*C_T_* method using *rpoA* as the control gene (amplified with rpoA-F and rpoA-R). All reactions were followed by a melting curve to determine amplicon purity.

### Phenotypic analyses.

For growth curve analyses, cultures of wild-type ECOR2 and the isogenic Δ*rpoS* mutant grown overnight were diluted to an optical density at 600 nm (OD_600_) of 0.01 in either sterile LB broth or M9 minimal medium containing 0.4% glucose. Two hundred microliters of each culture was transferred into a clear, flat-bottomed, 96-well microplate. At least six technical replicates were included per experiment. A VersaMax microplate reader (Molecular Devices, LLC, Sunnyvale, CA) was used to measure the OD_600_ at 20-min intervals in microplates maintained at 37°C with regular shaking over a 20-h time course.

To assess the ability of the Δ*rpoS* mutant to withstand stress *in vitro*, cultures of the Δ*rpoS* mutant and its wild-type parent strain grown overnight were diluted 1:100 in sterile LB broth and grown to mid-log phase (OD_600_ of ∼0.5 to 0.6). For membrane stress experiments, 500 μl of each culture was then treated with either 4, 6, or 8 μg/ml polymyxin B or water (as a control) and incubated for 1 h. Polymyxin B binds lipopolysaccharide to destabilize the membrane of Gram-negative bacteria (24). For pH stress assays, bacterial cultures were centrifuged at 4,000 × *g* for 5 min, and 1 ml of cells was then centrifuged a second time and resuspended in LB broth with a pH of 2.5 (adjusted with HCl). Cells were then incubated without shaking at 37°C for 15, 30, 60, and 90 min. Surviving cells were enumerated by serial plating on LB agar.

The metabolic properties of wild-type and Δ*rpoS* mutant ECOR2 were assessed with Biolog PM1 plates according to the manufacturer’s instructions. Bacteria grown overnight on LB agar were resuspended in Biolog inoculating fluid to a final OD_600_ of 1.0. Each well of the Biolog plate was inoculated with 100 μl of the cell suspension and incubated in a BioTek kinetic plate reader (BioTek Instruments, Winooski, VT) for 24 h. The OD_595_ was used to measure the reduction of tetrazolium violet dye every 15 min. The area under the curve (AUC) of the OD_595_ values was calculated as a measure of bacterial oxidation for each carbon source tested and plotted over time.

### HIO generation and experimentation.

HIOs (13, 16) were generated as described previously (14) and maintained in medium containing epidermal growth factor (EGF), Noggin, and R-spondin (ENR medium [see reference 15]) in Matrigel (8 mg/ml) without antibiotics prior to microinjection experiments. Bacterial cultures for microinjection were prepared by incubating wild-type ECOR2 and the Δ*rpoS* mutant overnight at 30°C with low shaking. The following day, cultures had reached an OD_600_ of ∼1.0 and were diluted 1:10 in sterile phosphate-buffered saline (PBS) and centrifuged for 10 min at 4,000 × *g*. Bacterial cells were then resuspended in sterile PBS. One hundred ninety microliters of this suspension was mixed with 10 μl of 4-kDa fluorescein isothiocyanate (FITC)-dextran suspended in PBS (2 mg/ml). FITC-dextran acted as a marker to ensure that HIOs were successfully microinjected and that the epithelial barrier remained intact (see below).

To introduce bacteria into the lumen of HIOs, microinjection was performed using thin-walled glass capillaries mounted on a Xenoworks micropipette holder with analog tubing, as previously described (11, 14). Each HIO was microinjected with approximately 10^4^ CFU of wild-type ECOR2 or the isogenic Δ*rpoS* mutant. After microinjection, HIOs were incubated for 1 h at 37°C. To remove bacteria introduced into the culture medium during the microinjection process, HIO culture medium was removed, and cultures were rinsed with PBS and washed with ENR medium containing 15 μg/ml gentamicin. HIOs were then washed again in PBS, and the medium was replaced with fresh antibiotic-free ENR medium. Successful microinjection was verified by visualizing the fluorescence of FITC-dextran in each HIO several hours after microinjection using an Olympus IX71 epifluorescence microscope. HIOs were considered successfully microinjected if an FITC signal was detectable. An absence of fluorescence was indicative of a loss of HIO structural integrity, and these organoids were excluded from further analyses.

Bacteria were enumerated in the HIO lumen as previously described (36). Briefly, HIO culture medium was removed from wells, and each organoid was placed into individual screw-cap tubes containing 300 μl PBS and 1.0-mm Biospec zirconia/silica beads (Fisher Scientific). HIOs were homogenized for 30 s in a Mini Bead Beater 8 instrument (Biospec Products). Viable bacteria were enumerated via serial plating of the homogenate on LB agar.

### Germ-free mouse colonization.

All animal experiments were performed with approval from the University Committee on Use and Care of Animals at the University of Michigan. Groups within an experiment were age matched to the greatest extent possible. Male and female germ-free Swiss Webster mice aged 6 to 9 weeks were obtained from a colony established and maintained by the University of Michigan Germ-Free Mouse Facility. Mice received sterile food, water, and bedding and remained bacteriologically sterile (except for the experimental E. coli strains) throughout the course of the experiments. Bacterial inocula were prepared as follows. Wild-type ECOR2 and the Δ*rpoS* mutant were grown in LB broth overnight at 30°C with low shaking. By the next morning, cultures had reached an OD_600_ of approximately 1.0. Cultures were then diluted 1:10 in sterile PBS and centrifuged at 4,000 × *g* for 10 min. The supernatant was discarded, and cells were resuspended in sterile PBS. Mice were inoculated via oral gavage with 100 μl of the bacterial suspension, which equated to about 10^6^ to 10^7^ CFU per mouse of each strain for monocolonization experiments and 10^6^ CFU per mouse of each strain administered together or sequentially (i.e., 24 h apart) for competition experiments, where indicated. To differentiate between wild-type and mutant growth, feces were collected and serially plated on LB agar with and without kanamycin sulfate (50 μg/ml). Feces were collected every other day for 7 days (monoassociation studies) or 14 to 15 days (competition experiments).

### Statistical analyses.

AUC values for Biolog assays and the corresponding heat map were generated using custom R scripts (https://github.com/barronmr/Biolog_AUC.git). All other analyses were performed using GraphPad Prism 8.3 (GraphPad Software, Inc.). When comparing two groups, a Welch’s unpaired *t* test was used for normally distributed data, and a Mann-Whitney U test was used for data that were not normally distributed. A *P* value of ≤0.05 was considered significant. Adobe Illustrator CC 2020 was used to arrange panels and generate the final figures.
