# Supplementary material for: The Lumen of Human Intestinal Organoids Poses Greater Stress to Bacteria Compared to the Germ-Free Mouse Intestine: Escherichia coli Deficient in RpoS as a Colonization Probe
Source: mSphere. 2020 Nov 11;5(6):e00777-20. doi: 10.1128/mSphere.00777-20 (PMC7657587; doi:10.1128/mSphere.00777-20)
Supplement: TABLE S1 [file mSphere.00777-20-st001.docx]

**Table S1**

| **Carbon Source** | **Wild-type** | $\Delta$***rpoS*** |
| --- | --- | --- |
| L-Arabinose | 11.3675 | 2.218 |
| N-Acetyl-D-Glucosamine | 10.892 | 3.243 |
| D-Saccharic acid | 0.2225 | 7.99425 |
| Succinic acid | 18.1765 | 14.408 |
| D-Galactose | 18.9065 | 8.851 |
| L-Aspartic acid | 21.06325 | 14.76125 |
| L-Proline | 4.79925 | 9.18325 |
| D-Alanine | 15.1535 | 10.60425 |
| D-Trehalose | 17.14925 | 10.6515 |
| D-Mannose | 14.0375 | 5.8075 |
| Galactitol | 0.12925 | 11.00175 |
| D-Serine | 18.68125 | 11.52275 |
| D-Sorbitol | 18.4395 | 7.98875 |
| Glycerol | 1.27825 | 12.55925 |
| L-Fucose | 19.70775 | 5.9375 |
| D-Glucuronic acid | 21.63375 | 9.988 |
| D-Gluconic acid | 25.94475 | 7.1635 |
| DL-a-Glycerol Phosphate | 13.265 | 8.2825 |
| D-Xylose | 16.0335 | 2.635 |
| L-Lactic acid | 19.3895 | 14.38625 |
| Formic acid | 0.54675 | 0.33925 |
| D-Mannitol | 14.55 | 5.85625 |
| L-Glutamic acid | 12.60075 | 0.818 |
| D-Glucose-6-Phosphate | 21.4825 | 15.68425 |
| D-Galactonic acid-g-Lactone | 15.52025 | 3.51625 |
| DL-Malic acid | 23.726 | 14.5435 |
| D-Ribose | 17.5555 | 9.54425 |
| Tween 20 | 1.0355 | 0.3835 |
| L-Rhamnose | 0.4 | 9.50925 |
| D-Fructose | 20.70925 | 4.78975 |
| Acetic acid | 7.5205 | 6.59825 |
| a-D-Glucose | 9.953 | 4.00975 |
| Maltose | 17.70025 | 12.0865 |
| D-Melibiose | 18.7635 | 6.62 |
| Thymidine | 21.87325 | 9.23925 |
| L-Asparagine | 20.405 | 13.959 |
| D-Aspartic acid | 0.1915 | 0.3075 |
| D-Glucosaminic acid | 0.663 | 0.425 |
| 1,2-Propanediol | 0.26525 | 0.137 |
| Tween 40 | 0.44825 | 0.09425 |
| a-Ketoglutaric acid | 2.82225 | 14.98675 |
| a-Ketobutyric acid | 0.1065 | 0.509 |
| a-Methyl D-Galactoside | 19.5615 | 13.6595 |
| a-D-Lactose | 13.50875 | 5.58725 |
| Lactulose | 17.0305 | 0.86225 |
| Sucrose | 0.4165 | 0.16575 |
| Uridine | 13.0465 | 5.0675 |
| L-Glutamine | 4.39975 | 2.62325 |
| m-Tartaric acid | 7.1435 | 0.14525 |
| D-Glucose-1-Phosphate | 27.145 | 12.922 |
| D-Fructose-6-Phosphate | 24.41075 | 10.86025 |
| Tween 80 | 0.9985 | 0.3935 |
| a-Hydroxyglutaric acid-g-Lactone | 0.71225 | 0.29475 |
| a-Hydroxybutyric acid | 2.23425 | 1.37775 |
| b-Methyl D-Glucoside | 1.903 | 2.191 |
| Adonitol | 23.22575 | 0.35075 |
| Maltotriose | 18.0155 | 10.29175 |
| 2`-Deoxyadenosine | 23.245 | 13.253 |
| Adenosine | 21.611 | 14.304 |
| Gly-Asp | 14.83425 | 3.655 |
| Citric acid | 0.207 | 0.1835 |
| m-Inositol | 0.8545 | 0.0325 |
| D-Threonine | 0.1175 | 0.01425 |
| Fumaric acid | 21.0515 | 13.509 |
| Bromosuccinic acid | 19.24975 | 6.9445 |
| Propionic acid | 0.23425 | 0.1405 |
| Mucic acid | 23.88575 | 8.779 |
| Glycolic acid | 0.33125 | 6.32125 |
| Glyoxylic acid | 0.0685 | 1.63 |
| D-Cellobiose | 1.643 | 0.42325 |
| Inosine | 23.30575 | 15.548 |
| Gly-Glu | 12.42725 | 1.8815 |
| Tricarballylic acid | 0.554 | 0.087 |
| L-Serine | 20.85225 | 13.0075 |
| L-Threonine | 13.349 | 8.58625 |
| L-Alanine | 14.857 | 8.82325 |
| Ala-Gly | 18.87675 | 13.99425 |
| Acetoacetic acid | 0.74475 | 0.85075 |
| N-Acetyl-D-Mannosamine | 4.4155 | 1.17 |
| Mono-methyl Succinate | 4.151 | 1.41375 |
| Methyl Pyruvate | 17.614 | 9.06825 |
| D-Malic acid | 21.43275 | 14.47625 |
| L-Malic acid | 23.30725 | 14.599 |
| Gly-Pro | 18.29875 | 8.85975 |
| p-Hydroxyphenyl Acetic acid | 0.63325 | 0.56225 |
| m-Hydroxyphenyl Acetic acid | 0.3805 | 0.12675 |
| Tyramine | 0.3555 | 0.043 |
| D-Psicose | 4.2975 | 0.19975 |
| L-Lyxose | 0.03025 | 0 |
| Glucuronamide | 0.1545 | 0.082 |
| Pyruvic acid | 19.00825 | 8.3875 |
| L-Galactonic acid-g-Lactone | 16.914 | 9.9615 |
| D-Galacturonic acid | 21.07075 | 7.73225 |
| b-Phenylethylamine | 0.102 | 0.9165 |
| Ethanolamine | 0.2335 | 0.23375 |
